# Understanding cachexia and muscle dysfunction in the context of metastatic breast cancer

**DOI:** 10.1007/s10555-026-10358-7

**Published:** 2026-07-17

**Authors:** Alastair A. E. Saunders, Robin L. Anderson, Paul Gregorevic

**Affiliations:** 1 Centre for Muscle Research, and Department of Anatomy and Physiology, The University of Melbourne, Parkville, VIC Australia; 2 Olivia Newton-John Cancer Research Institute, Heidelberg, VIC Australia; 3 School of Cancer Medicine, La Trobe University, Bundoora, VIC Australia; 4 Department of Clinical Pathology, The University of Melbourne, Parkville, VIC Australia; 5 Department of Neurology, The University of Washington School of Medicine, Seattle, WA USA

**Keywords:** Breast cancer, Metastasis, Muscle, Cachexia, Sarcopenia, Models

## Abstract

Cancer cachexia significantly impacts the quality of life of cancer patients, predicting therapy response and survival. Cachexia prevalence and severity increases over the course of disease progression, affecting up to 80% of patients with metastatic cancer. Despite this, cachexia is relatively understudied in the context of metastatic breast cancer, despite it affecting a significant proportion of patients with this common cancer. In this review we highlight the relative prevalence of cachexia in breast cancer, with 25% of all patients and up to half of those with metastatic disease impacted. Given the metastatic distribution pattern of breast cancer (*e.g.* high rates of bone metastases), we reviewed the unique systemic and local muscle cues leading to cachexia in this context and have reviewed areas for further investigation. Furthermore, anti-cancer therapies also contribute to cachexia progression. Recently, novel mouse models for study of cachexia in breast cancer have been developed. These models can further the field’s understanding of cachexia in metastatic cancer patients and aid in the development of anti-cachectic agents. However, it is important that these models accurately recapitulate human disease. In this review we provide an appraisal of the relative utility of the currently available models. Overall, this review highlights that understanding cachexia in the context of breast cancer and metastasis may uncover novel mechanisms to target this condition, thereby improving the quality of life and survival prospects of metastatic cancer patients.

**Supplementary Information:**

The online version contains supplementary material available at 10.1007/s10555-026-10358-7.

## Introduction

The loss of muscle and fat mass caused by cancer is a significant determinant of the quality of life of cancer patients, and for many patients, contributes to premature death [1]. This loss of functional muscle and fat mass, known as cachexia, is caused by systemic inflammation, an increased metabolic rate, malnutrition and secretion of cytokines from tumors [2]. Cachexia significantly burdens cancer patients, with one study reporting that as cachexia progresses, cancer patients have reduced feelings of well-being and increased distress [3]. These symptoms are likely driven by a loss of strength and increased fatigue, which can limit mobility and the ability to perform independent tasks. Patients with metastatic cancer have a higher prevalence of cachexia than those with earlier-stage disease [4, 5]. Breast cancer has traditionally been understudied in the context of cachexia, as the prevalence of cachexia is not as high as in some other cancers [2]. However, many breast cancer patients will develop cachexia over the course of disease progression, underscoring the need to understand its underlying mechanisms in this context.

As well as impacting the quality of life of cancer patients, cachexia reduces survival prospects. A meta-analysis of six studies with 5497 subjects found that sarcopenia in breast cancer patients was associated with higher mortality risk compared to those without cachexia [6]. This observation is not unique to breast cancer, with data collated in other meta-analyses suggesting that the presence of sarcropenia is negatively associated with survival in lung [7], gastric [8] and colorectal cancers [9]. The presence of cachexia in patients also predicts increased risk of complications following tumor resection surgery [10]. These observations suggest that clinicians should screen patients for cachexia to better personalize their cancer treatment. Furthermore, preclinical research should investigate mechanisms of cachexia to guide development of effective therapies and interventions.

This review will assess the prevalence of cachexia in patients with breast cancer relative to other cancer types. We will review the current literature describing the systemic cues contributing to muscle and fat loss in breast cancer, and the local muscle responses to these unique systemic cues that lead to cachexia. Recently, preclinical research has utilized multiple animal models to study this condition. We provide an appraisal of current models for studying breast cancer cachexia.

## Prevalence and incidence of breast cancer cachexia

**Fig. 1:**
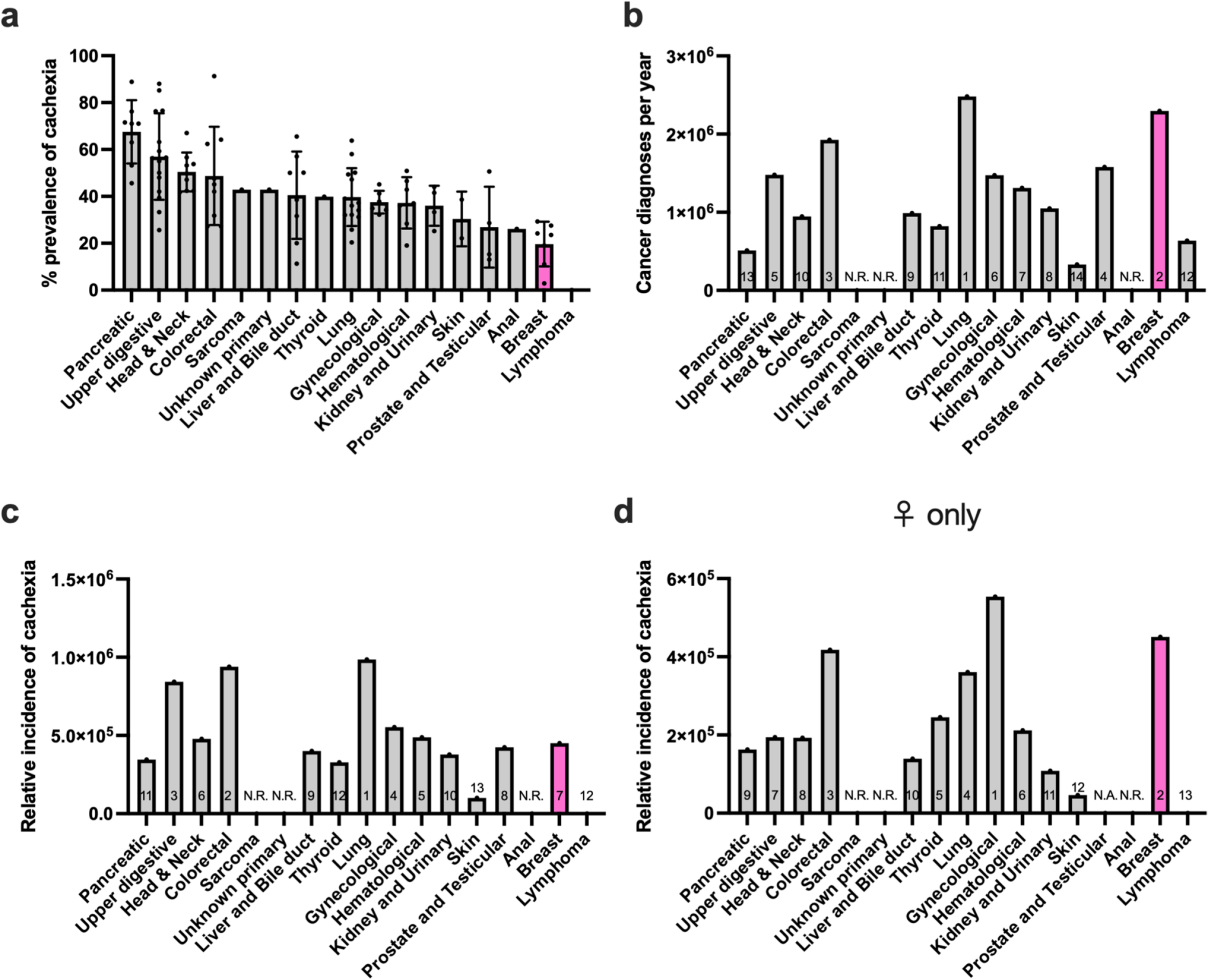
Estimated prevalence of cachexia by cancer type. (**A**) The percent prevalence of cachexia by cancer type (mean±SD), collated from [14–27], each data point represents a different study. (**B**) The new cases of cancer in 2022 (worldwide), with data from [42]. (**C**) Estimation of cachexia frequency by multiplying the prevalence of cachexia by the new cases of cancer each year, and (**D**) estimation of cachexia frequency in females. Numbers in bar graph represent rating of most frequent (1) to least frequent (14). N.R. = not reported; N.A. = not applicable

Some studies have reported that cachexia does not occur frequently in breast cancer [11, 12]. To clarify this, we estimated the clinical prevalence of cachexia associated with breast cancer compared to the frequency of cachexia across other cancer types. A search of the literature in PubMed was conducted using the following search terms: ((cachexia[tiab] OR "wasting syndrome"[tiab]) AND (cancer[tiab] OR carcinoma*[tiab] OR neoplasm*[tiab] OR malignan*[tiab]) AND (incidence[tiab] OR prevalence[tiab] OR epidemiolog*[tiab])) AND ("2010/01/01"[Date—Publication]: "2025/06/01"[Date—Publication]) AND (humans[Mesh]) AND (english[lang]) NOT ("animals"[Mesh] NOT "humans"[Mesh]) NOT ("molecular"[tiab] OR "mechanism"[tiab] OR "pathway"[tiab] OR "biochemical"[tiab]) NOT ("case report"[Publication Type]). This search strategy led to 294 results, of which 219 were excluded for not being relevant, 12 were excluded for not distinguishing cancer types, 37 were excluded for using a definition of cachexia not consistent with the consensus definition as defined by Fearon and colleagues [13], resulting in 27 studies (across 26 papers) being included in the analysis [14–39] (Table 1; Supplemental File). The prevalence of cachexia differed among primary cancer sites (Fig. 1a). Pancreatic (67.6%), upper digestive (60.0%), head and neck (50.4%), and colorectal (48.7%) were examples of cancers that were more frequently reported as associated with cachexia. By comparison, cachexia was indicated as occurring in approximately 19.6% of those with breast cancer (Fig. 1a). The prevalence is likely to be higher in metastatic breast cancer, with one study finding that 36.1% of patients presenting with sarcopenia (as defined by skeletal muscle index < 39 cm^2^/m^2^; where skeletal muscle index is muscle area at the L3 region of the spine in cm^2^) at the time of diagnosis of metastasis, and 45.5% at initiation of third-line treatment [40]. These data indicate that the prevalence of cachexia in metastatic breast cancer is higher than in early-stage breast cancer, however there are limited clinical data stratifying these patient populations.

**Table 1:** Tabular summary of incidence of cachexia by cancer type

Author, Date	PMID	Country	Upper digestive	Head & Neck	Lung	Blood	Gyneco-logical	Colo-rectal	Anal	Breast
Pressoir et al. 2010 [14]	20160725	FRA	63.2	56.6	49.4	42.9	45.1	47.5	-	24.2
Muscaritoli et al. 2017 [16]	29108370	ITA	85.2	47.1	47.2	-	-	64.2	-	27.5
Li et al. 2018 [17]	30485919	CHN	59.3	67	32.1	36	34.2	45.1	-	19
Gilmore et al. 2025 [18]	40323088	USA	-	-	58	-	-	-	-	-
Takaoka et al. 2024—systemic review [19]	39127425	Various	25.6	41.8	32	37	37.8	-	-	-
Takaoka et al. 2024—meta analysis [19]			39.4	49.8	34	46.5	-	-	-	-
Rich et al. 2021. [20]	34555519	USA	-	-	-	-	-	-	-	-
Zhang et al. 2025 [21]	40241395	Various	50	-	-	-	-	-	-	-
March et al. 2022 [22]	35623801	Various	-	-	-	-	-	-	-	-
Anker et al. 2019 [23]	30920776	Various	33.3	42.3	37.2	28.4	32.2	31.8	-	23.5
Poisson et al. 2021 [24]	34519440	FRA	76.3	53.8	63.8	50.9	41.2	62.4	-	29.3
Shukuya et al. 2023 [25]	36905129	JPN	-	-	20.4	-	-	-	-	-
Vagnildhaug et al. 2018 [26]	29,274,028	NOR	42	-	36	19	-	-	-	11
Kwon et al. 2017 [27]	28,000,343	KOR	-	53.2	-	-	-	-	-	-
Zhang et al. 2024 [28]	38,788,267	Various	-	-	39	-	-	-	-	-
Namikawa et al. 2022 [29]	35322296	JPN	55	-	-	-	-	-	-	-
Mitsunaga et al. 2020 [30]	32103356	JPN	-	-	-	-	-	-	-	-
Dijksterhuis et al. 2021 [31]	33418527	NED	48	-	-	-	-	-	-	-
Latenshein et al. 2020 [32]	33107709	NED	-	-	-	-	-	-	-	-
Olaechea et al. 2023 [33]	37099735	USA	56.3	-	-	-	-	27.2	-	-
Gannavarapu et al. 2018 [34]	29466074	USA	56.5		31.2	-	-	27.2	26.1	-
Shibata et al. 2020 [35]	33067699	JPN	-	-	-	-	-	91.3	-	-
Nemer et al. 2017 [36]	28902785	USA	-	-	-	-	-	-	-	-
Sun et al. 2015 [37]	26317149	CHN	76.5	42.4	25.8	-	34.9	42	-	2.9
Fukahori et al. 2021 [38]	32361831	JPN	88	-	-	-	-	-	-	-
Katsushima et al. 2025 [39]	39825794	JPN	-	-	50.1	-	-	-	-	-

Author, Date	PMID	Pancreatic	Prostate	Urinary	Unknown primary	Liver & Bile Duct	Melanoma	Thyroid	Sarcoma	Lymphoma
Pressoir et al. 2010 [14]	20160725	-	-	-	-	-	-	-	-	-
Muscaritoli et al. 2017 [16]	29108370	76.2	-	41.5	42.9	65.5	-	-	-	-
Li et al. 2018 [17]	30485919	63	28.6	33.3	-	31.6	-	-	-	-
Gilmore et al. 2025 [18]	40323088	-	-	-	-	-	-	-	-	-
Takaoka et al. 2024—systemic review [19]	39127425	-	-	-	-	20	-	-	-	-
Takaoka et al. 2024—meta analysis [19]		-	-	-	-	11.3	-	-	-	-
Rich et al. 2021. [20]	34555519	-	-	-	-	57	-	-	-	-
Zhang et al. 2025 [21]	40241395	-	-	-	-	-	-	-	-	-
March et al. 2022 [22]	35623801	-	-	-	-	38.5	-	-	-	-
Anker et al. 2019 [23]	30920776	45.6	15.3	25.2	-	50.1	22.1	39.9	-	-
Poisson et al. 2021 [24]	34519440	-	50.6	44	-	-	38.6	-	-	-
Shukuya et al. 2023 [25]	36905129	-	-	-	-	-	-	-	-	-
Vagnildhaug et al. 2018 [26]	29,274,028	-	13	-	-	-	-	-	-	-
Kwon et al. 2017 [27]	28,000,343	-	-	-	-	-	-	-	-	-
Zhang et al. 2024 [28]	38,788,267	-	-	-	-	-	-	-	-	-
Namikawa et al. 2022 [29]	35322296	-	-	-	-	-	-	-	-	-
Mitsunaga et al. 2020 [30]	32103356	71	-	-	-	-	-	-	-	-
Dijksterhuis et al. 2021 [31]	33418527	-	-	-	-	-	-	-	-	-
Latenshein et al. 2020 [32]	33107709	71	-	-	-	-	-	-	-	-
Olaechea et al. 2023 [33]	37099735	-	-	-	-	-	-	-	-	-
Gannavarapu et al. 2018 [34]	29466074	53.2	-	-	-	-	-	-	-	-
Shibata et al. 2020 [35]	33067699	-	-	-	-	-	-	-	-	-
Nemer et al. 2017 [36]	28902785	71.5	-	-	-	-	-	-	-	-
Sun et al. 2015 [37]	26317149	88.9	-	-	-	50	-	-	42.9	0
Fukahori et al. 2021 [38]	32361831	-	-	-	-	-	-	-	-	-
Katsushima et al. 2025 [39]	39825794	-	-	-	-	-	-	-	-	-

Numbers indicate prevalence of cachexia expressed as a percentage. Only studies that defined cachexia according to the Fearon definition were included [13].- = not reported in study

It is important to note that the papers used in this analysis included data primarily from Western and Asian countries, with Japan (6 studies) USA (5 studies), France (3 studies), China, Netherlands (both 2 studies), Italy, Norway and Korea (all 1 study, with 6 studies using data from multiple populations) used to gather prevalence of cachexia. It is unclear whether cachexia prevalence in these breast cancer populations may be low due to early detection from screening (Fig. 1a). For instance, in Australia 5% of breast cancer patients are diagnosed at stage IV, compared to 18% for colon and 42% for lung cancers [41]. In countries with limited access to early screening of breast cancer, the prevalence of cachexia may be higher. It is important to note that lifestyle, heritage, breast cancer subtype or breast cancer stage were not considered in these analyses, thereby impacting cachexia prevalence and severity.

We collated the number of new cases by cancer type from data collated by the World Cancer Research Fund (Fig. 1b [42]). We next estimated the incidence of cachexia at the population level by multiplying the total number of cancer diagnoses per year (Fig. 1b) [42] (as an approximation of total cancer incidence) by the prevalence of cachexia in each cancer type (Fig. 1a). Lung, colorectal, upper digestive, gynecological, hematological, head and neck, followed by breast cancer, exhibited the highest incidence when factoring in total cancer diagnoses (Fig. 1c). These data indicate that although the prevalence of cachexia in breast cancer may not be as high as other cancers, the total number of breast cancer patients with cachexia is considerable.

Given breast cancer largely affects females, we then multiplied the incidence of cachexia (Fig. 1a), by the number of cancer diagnoses in females only (Fig. 1d). In females, the most frequent cases of cachexia were associated with gynecological and breast cancers (Fig. 1d). However, this estimate of cachexia takes into account the frequency of cachexia across both sexes, corrected for cancer diagnoses only in females. Very few papers stratify cachexia incidence by sex and cancer type, so it is difficult to accurately determine cachexia incidence only in females. There are differences in prevalence and pathogenesis of cachexia between the sexes [43, 44]; therefore, it is conceivable that the prevalence of cachexia in different primary cancers varies by sex. Nonetheless, in females, gynecological and breast cancers contribute a relatively large proportion of cachexia cases. Assessment of cachexia prevalence, accounting for the incidence of individual cancer types highlights the need to study cachexia in breast cancer.

We also searched for studies that reported the prevalence of cachexia in metastatic *vs.* non-metastatic breast cancer patients (as presented in Table 2). Due to the lack of papers reporting the prevalence between these patient populations, we extended our search beyond the limitation of 2010–2026, and also included papers citing sarcopenia or cachexia. Dewys and colleagues reported weight loss in 49% of metastatic breast cancer patients compared to 31% in non-metastatic patients [45]. It is important to note that this study was conducted more than 4 decades ago, with advancements in both treatment and diagnosis occurring since then; therefore, the prevalence reported in this study may not reflect current rates of cachexia. However, a similar difference was reported in another study that used > 5% weight loss or BMI < 20 with weight loss of 2% to 5% as the cutoff for cachexia with 27.5% and 10.8% rates of cachexia in metastatic and non-metastatic breast cancer patients, respectively [16]. Other reported prevalence of sarcopenia in metastatic breast cancer patients are 36.1% and 41.6% [40, 46] (Table 2). From the limited number of papers that report on cachexia prevalence in metastatic breast cancer, the prevalence appears to range between 30–50% (Table 2). Comparatively, in other cancer types, cachexia prevalence in metastatic cancer patients ranges from 40–85% depending on the cancer type [16]. Muscaritoli and colleagues reported the prevalence of cachexia to be particularly high in metastatic GI (85.2%) pancreatic (76.2%), liver (65.5%) and colorectal (64.2%) cancer patients [16].

**Table 2:** Summary of prevalence of cachexia in metastatic breast cancer

Author, Date	PMID	Cachexia rate in early breast cancer	Cachexia rate in metastatic breast cancer	Cachexia definition
Dewys et al., 1980 [45]	7424938	31	49	Weight loss over 2 months
Muscaritoli et al., 2017 [16]	29108370	10.8	27.5	> 5% weight loss, or BMI < 20 with 2–5% weight loss over 6 months
Camilleri et al., 2024 [40]	38908032	N.R	36.1	Weight loss of ≥ 5% within one month, or ≥ 10% within six months, or ≥ 10% of the usual body weight, or BMI < 18.5 kg/m^2^, or < 22 kg/m^2^ in patients 70 yrs or older
Jang et al., 2025 [46]	40446428	N.R	41.6	SMI cutoffs between 38–41 cm^2^/m^2^

*N.R.* Not reported, *SMI* Skeletal muscle index, *BMI* Body mass index

## Clinical case studies reporting breast cancer cachexia

Cachexia has been reported in clinical case studies of breast cancer patients [11, 12]. One such case study assessed subcutaneous fat, visceral fat, abdominal muscle, and paraspinal muscle mass through cancer progression by computed tomography (CT) scans, and found that cross sectional area (CSA) of these tissues decreased over time as the disease progressed [11]. Another case study of a breast cancer patient with bone metastases found that subcutaneous fat, abdominal muscle, and paraspinal muscle mass, but not visceral fat area correlated with metastatic tumor area [12]; highlighting that metastatic tumor progression may accelerate cachexia. Another case study highlighted frailty, weakness and a loss of appetite in a patient with breast cancer untreated for 3 years [47]. These case studies highlight that over the course of breast cancer progression, muscle wasting can significantly impact the quality of life of cancer patients.

Clinical evidence of cachexia in breast cancer has also been demonstrated in a study reporting smaller muscle fiber CSA in biopsies from individuals with breast cancer relative to cancer-free individuals [48]. This reduction in muscle size was associated with defects in mitochondria, measured by electron microscopy from muscle biopsies [48]. Functionally, forearm strength was also reduced in breast cancer survivors [49]. Additionally, transcriptomic profiling of skeletal muscle from early-stage breast cancer patients revealed impairments in oxidative phosphorylation and mitochondrial function, suggesting that muscle dysfunction may begin early in the disease course [50]. Furthermore, a longitudinal study of patients with non-metastatic breast cancer found that 84% of these individuals had either reduced muscle function or size over time [51]. A study of breast cancer patients undergoing chemotherapy reported that muscle CSA, muscle capillarization, and citrate synthase activity – a measure of muscle aerobic capacity – declined during 16 weeks of treatment [52]. Aerobic training in combination with high-intensity interval training restored the reduction in CSA in only type I fibers (oxidative) and resistance training with high-intensity interval training increased CSA in both type I and type II (fast twitch) fibers [52]. The authors also found that mitochondrial complex I, II and IV protein expression was elevated only following aerobic training and high-intensity interval training [52], consistent with the increase in CSA only in slow/oxidative muscle fibers. These case studies of cachexia in breast cancer patients further highlight the impact of cachexia on some breast cancer patients. Preclinical studies have complemented these observations, where patient-derived xenografts of different breast cancer subtypes in mice caused muscle dysfunction and fatigue [53, 54].

## Systemic drivers of cachexia in breast cancer

### Secretion of cytokines and extracellular vesicles from the primary tumor

**Fig. 2:**
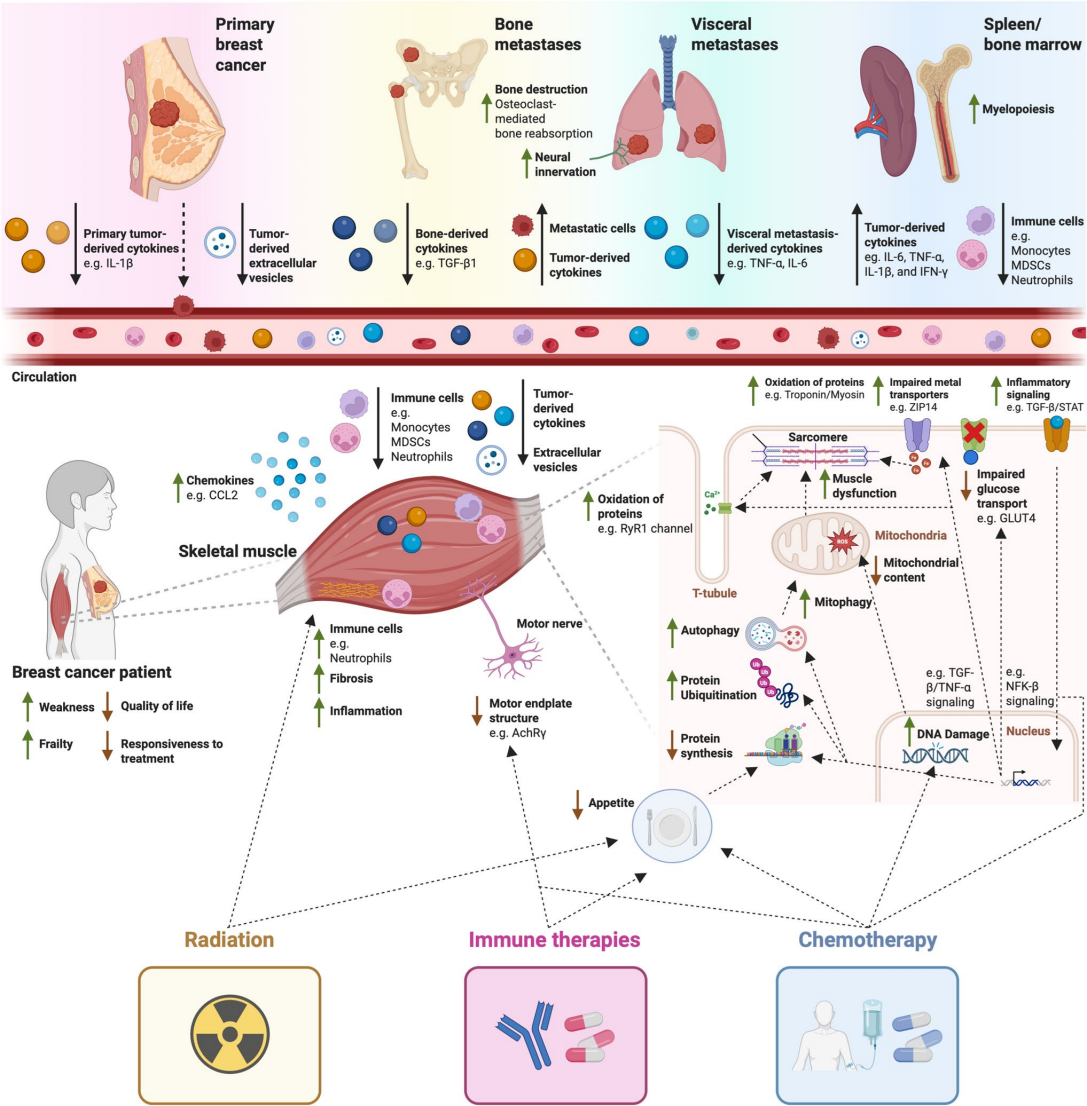
Mechanisms of tissue crosstalk and cachexia in breast cancer. In breast cancer, there is evidence to suggest that cues from the primary tumor, as well as distal metastases contribute to cachexia progression. Primary tumors (top left) secrete cytokines and EVs. Tumor cells are released from the primary tumor into the circulation. Tumor derived cytokines and CTCs disseminate to metastatic niches (top center) to form metastases. Metastatic lesions cause myelopoiesis in the bone marrow and spleen (top right) leading to increased circulating immune cells. Primary tumor and/or metastatic derived cytokines as well as immune cells (through increased chemoattraction) increase in abundance in skeletal muscle (center). These inflammatory cytokines impact neuromuscular health. These inflammatory cytokines lead to increased inflammatory signaling (center right), which results in alterations in proteostasis, impaired metal transporters, glucose transport, mitochondrial content, and ultimately muscle dysfunction. Chemotherapy, immune therapies and radiation therapies (bottom) disrupt muscle health through inflammation (center right), DNA damage (center right), mitochondrial dysfunction (center right) defects to the motor nerve (center), and reduced appetite (bottom). AchRγ = Acetylcholine receptor gamma; CCL2 = C–C motif chemokine ligand 2; CTC = circulating tumor cell; EV = extracellular vesicle; MDSC = myeloid-derived suppressor cell; IFN = interferon**;** IL = interleukin; ROS = reactive oxygen species; RyR1 = ryanodine receptor 1; TGF-β = transforming growth factor-beta; TNF-α = tumor necrosis factor-alpha. Created in BioRender. Saunders, A. (2026) https://BioRender.com/43ks0r3

There is evidence that primary tumor-derived factors may contribute to cachexia in breast cancer (Fig. 2). Kumar and colleagues demonstrated that secreted cytokines from primary breast tumors can induce cachexia [55]. In this study, MCF-7 human breast cancer cells were modified to produce a secreted form of interleukin 1a (IL-1a), and when engrafted into nude mice, resulted in a progressive loss of body mass over the course of six weeks to a greater extent than parental tumors. Incubation of rat muscles with IL-1 increased muscle protein breakdown [56], while twice daily delivery of IL-1a to Wistar rats induced anorexia and cachexia [57]. Analysis of transcriptional datasets showed that breast cancers had increased expression of pro-cachectic factors, including *IL-1β* [58]. IL-1β is a pro-inflammatory cytokine that has been linked to cachexia promotion [59]. It is produced by myeloid cells, and stimulates IL-6 expression [60] that is involved in the inflammatory response in muscle to cachexia and has emerged as a potential therapeutic target to reduce cachectic progression [61, 62]. Another study highlighted the role of systemic IL-1β in a study of Lewis lung carcinoma (LLC)-bearing mice, showing that it is elevated in the hypothalamus and is associated with muscle protein breakdown [63]. Analysis of the expression of known cancer cachexia inducing factors in RNA sequencing data from breast, colon, esophageal, head and neck, acute myeloid leukemia, liver, lung, pancreatic, prostate, rectal and stomach cancers revealed distinct expression profiles of these cytokines in different cancer types [64]. For example in breast cancer, *CXCL9*, *CXCL10* and *INHBA* (Activin A) were notably increased [64], indicating that secretion of these cytokines may contribute to cachexia in breast cancer. These cytokines have been well characterized to induce cachexia. For instance, delivery of viral vectors expressing Activin A, either systemically or directly to muscle promotes muscle wasting and weakness in mice [65, 66].

Extracellular vesicles (EVs) secreted by primary tumors may be involved in breast cancer-induced cachexia (Fig. 2). Hu and colleagues isolated EVs from 4T1, MCF-10 and MDA-MB-231 conditioned medium (CM) and found that EVs derived from 4T1 and MDA-MB-231 cells reduced both inguinal and epi-gonadal white adipose tissue in mice [67]. No difference in fat deposition was seen with EVs from MCF-10 non-tumorigenic breast cancer cells [67]. The authors attributed the fat loss seen in the triple-negative 4T1 and MDA-MB-231 lines to cancer cell-secreted exosomal miR-204-5p that induced hypoxia-inducible factor 1 A (HIF1A) in white adipose tissue [67]. HIF1A altered the mitochondrial content and thermogenesis to increase lipolysis and fat loss. Whether EV secretion from breast cancer cells results in muscle loss is not as well established and is a potential avenue for future research.

### Secretion of cytokines from visceral metastases

The progression of metastasis has been linked to cachexia severity and progression [68]. There are parallels between steps of the metastatic cascade, and the genes involved in the various steps of metastasis that are related to cachexia development [68, 69]. For instance, inflammatory cytokines such as tumor necrosis factor-α (TNF-α) and IL-1β promote invasion in breast cancer [70] (Fig. 2). TNF-α is well characterized inducer of cachexia in non-breast cancer models [71]. The source of TNF-α in cachexia is not fully understood; however, it is hypothesized to be secreted from tumor cells and macrophages [72]. Macrophage infiltration into muscle has been described in cachexia [73] and may be the source of TNF-α that drives cachexia. Other inflammatory cytokines such as IL-6 are also key regulators of metastasis in breast cancer [74]. IL-6 signaling is well characterized to promote cachexia, acting both to both reduce fat mass [61, 66], and directly on the muscle to drive atrophy [75, 76].

These reports indicate that there may be specific cues secreted from metastatic tumors that cause or exacerbate cachexia [77]. The role of visceral metastasis independent from primary tumors has been demonstrated in preclinical studies where the primary tumor is resected and mice subsequently develop metastases and muscle and fat loss, indicative of cachexia [78, 79]. In non-breast cancer models, experimental induction of liver metastasis following intrasplenic injection of colon cancer cells resulted in decreased muscle mass, as well as increased mRNA expression of *Trim63*/*Murf-1*, *Fbox32*/*Atrogin-1*, and *Il-6* in the tibialis anterior muscle [80]. It is therefore evident that inflammatory cytokines secreted from visceral metastatic lesions likely contribute to cachectic progression in breast cancer as well; however, there are limited studies demonstrating the precise mechanisms and cytokines secreted by visceral metastases to drive cachexia.

### Osteolytic secretion of cytokines from bone metastases

Rates of bone metastases are particularly high in breast cancer [81], and bone metastatic derived cytokines may contribute to cachexia progression (Fig. 2). Recently, bone-to-muscle crosstalk has been described as a regulator of skeletal muscle health [82, 83]. Waning and colleagues completed intracardiac injections of 100,000 MDA-MB-231 human cancer cells into nude mice to induce bone metastasis, resulting in lowered lean mass and reduced muscle function [84]. Analysis of post-translational protein modifications by unbiased mass spectrometry of muscles from these mice, and from breast cancer patients with metastatic disease revealed that proteins involved in muscle contraction were highly oxidized [84]. Notable from these analyses were sarcomeric proteins such as tropomyosin, myosin and the ryanodine receptor 1 (RyR1) Ca2 + release channel. These results were largely replicated in other osteolytic models, including A549 (lung cancer), PC3 (prostate cancer), JJN3 (multiple myeloma) as well as mixed osteolytic/osteoblastic bone metastases (RWGT2 lung cancer and MCF-7 breast cancer) [84]. Pharmacological reduction of RyR1 leak through subcutaneous administration of S107 partially improved grip strength and increased specific force back to normal levels [84]. Administration of transforming growth factor-β (TGF-β) receptor I kinase inhibitor SD-208 (by daily oral gavage), a TGF-β neutralizing antibody, or reducing TGF-β release from the bone matrix through the bisphosphonate, zoledronic acid (by subcutaneous injection), also improved muscle function [82, 83].

As the immune system is an important regulator of metastasis [85] and skeletal muscle health [86], the study by Waning and colleagues [84] was followed up by Regan and colleagues who completed intra-tibial injections of 4T1 murine breast cancer cells to simulate bone tumor colonization [87]. This experimental setup resulted in reduced fat and muscle mass after four weeks [87]. The authors attributed these changes to impaired RyR1 signaling, consistent with their previous work [84]. An advantage of the intratibial model, is that tumor formation was likely exclusively in the bone microenvironment, and therefore muscle impairments were driven solely by secretion from the bone environment. In addition to muscle wasting, breast cancer and bone metastases have been reported to induce lipolysis of fat tissue through mechanisms such as secretion of TGF-β, TNF-α, IL-6 and parathyroid hormone-related protein (PTHrP) from both primary and metastatic lesions as reviewed by Soni and colleagues [88].

### Neuro-inflammation associated with perineural invasion and tumor innervation

Imoto and colleagues described that body mass and BMI were lower in pancreatic cancer patients whose tumors were highly innervated [89]. To experimentally induce perineural invasion, the same group injected pancreatic tumor cells into the sciatic nerve of mice, which resulted in astrocyte activation leading to neuroinflammation and muscle wasting [89]. This effect could be reversed by ligating the nerve [89]. Furthermore, in a murine lymphoma cachexia model, sympathectomy through delivery of 6-OHDA reduced muscle wasting, suggesting a role for the sympathetic nervous system in cachexia progression [90]. Perineural invasion and tumor innervation has been observed in multiple cancers, and the density of nerves correlates with more aggressive cancers of the prostate, colon and rectum, head and neck, breast, pancreas, stomach and lung [91]. As many of these highly innervated tumors are associated with significant cachexia (Fig. 1) it is conceivable that the ability of tumors to hijack the nervous system may presently be an under-appreciated contributor to cachexia, particularly when highly metastatic cancers disseminate and seed into extensively innervated tissues. Innervation has been described in primary breast cancer [92], although there is no research to-date exploring whether this phenomenon contributes to cachexia. To date, most research on neuronal regulation of cancer has focused on primary cancers, where there is emerging evidence that innervation can influence metastatic colonization [93–95]. In the context of breast cancer, it may be more likely that innervation becomes a factor in cachexia when metastatic secondary tumors develop in other organs known to have stronger association with cachexia. Potentially, the role of perineural invasion and tumor innervation in both primary and metastatic breast lesions in promoting cachexia is an area for future research.

## Muscle-derived regulators of cachexia in breast cancer

### Decreased muscle protein synthesis

Following the systemic cachexia inducing cues in breast cancer, there is a cascade of events that occur in muscle that result in muscle atrophy and weakness (Fig. 2). One such response is a reduction in protein synthesis following inflammatory signaling in muscle (Fig. 2). We have reported differences in muscle protein synthesis rates between models of breast cancer, with the more severely cachectic EMT6.5 tumor-bearing mice displaying a reduction in puromycin-labelled proteins relative to muscle from 4T1.2 tumor-bearing mice, indicating a reduction in synthesis of new proteins [79]. Impairments in protein synthesis have been reported in other cachectic models, for instance in mice bearing murine MAC16 colon adenocarcinoma, as measured by L-[4-3H]phenylalanine labelling of proteins in gastrocnemius muscles [96]. In colon-26 (C26) tumor-bearing mice, puromycin labelled proteins were also decreased, further highlighting impaired protein synthesis as a hallmark of muscle cachexia [97, 98].

A key signaling pathway contributing to protein synthesis is the mammalian target of rapamycin (mTOR)- AKT pathway that is impaired in cancer cachexia. mTOR activity is reduced in both the C26 and LLC tumor-bearing mouse models of cachexia, as shown by decreased expression of phosphorylated AKT and pS6-Kinase and a suppressed rate of protein synthesis [97]. Furthermore, cachexia may induce anabolic resistance, as evidenced by a decreased capacity of Apc^Min/+^ mice to activate mTOR signaling after administration of a glucose bolus [99]. In a comparative study using breast cancer models, phosphorylated AKT was reduced only in the more severely cachectic EMT6.5 model compared to 4T1.2 tumor bearing mice. However, phosphorylated mTOR and 4EBP-1 were reduced to a similar extent in both models [79]. In summary, the perturbation of the mTOR pathway in breast cancer cachexia is consistent with observations in other cancer types.

### Increased muscle protein degradation

In addition to impairments in protein synthesis, protein degradation is typically elevated in settings of cancer cachexia [100] (Fig. 2). As there are few techniques that reliably measure protein breakdown and proteasome activity, mRNA and protein expression of E3 ligases are often used as surrogate measures of protein degradation [101]. Atrogin-1 and Murf-1 protein levels in C2C12 cells and in muscle from 4T1 tumor-bearing mice were elevated as measured by western blot and immunolabelling, respectively [102]. In an experimental model of intratibial 4T1 bone colonization, transcript levels of *Fbox32* and *Trim63* were elevated in muscle [87], demonstrating that muscle deficits occur following localized bone metastasis. Similarly, in an orthotopic 4T1 model *Fbox30*, *Fbox31*, *Fbox32*, and *Trim63* were all elevated in tibialis anterior and diaphragm muscles as measured through RT-qPCR [78]. This gene signature was consistent with another study of Bard1-deficient breast tumor-bearing mice [103] and EMT6.5 orthotopic tumor-bearing mice [79].

Another mechanism of muscle protein breakdown is autophagy. In EMT6.5 breast tumor-bearing mice, *Gabarapl1* (which contributes to autophagosome maturation, and lysosome fusion) mRNA was elevated, with a trend for an increase in *Map1lc3b* (which encodes lc3b which is involved in autophagosome formation) and *Sqstm1* (which encodes p62) in this model [79]. Lc3b protein expression as measured by western blot was also elevated in C2C12 muscle cells treated with conditioned medium (CM) from 4T1 cells [102]. Increased protein expression of these aforementioned autophagy markers was also observed in immunocompromised mice administered MDA-MB-231 human breast cancer cells by intracardiac injection [104]. These changes have been observed in other cancers; for instance, in muscle from gastric cancer patients, Beclin mRNA and protein expression – which is associated in autophagy initiation – was elevated in cachectic vs weight stable patients [105]. Furthermore, mRNA and protein levels of LC3B, were elevated in both weight-losing and weight-stable cancer patients relative to healthy; with LC3B protein expression elevated more in weight-losing patients relative to weight-stable patients [105]. p62 is another marker of autophagy, and a similar trend was observed in both protein and mRNA in the same study [105]. These data indicate that autophagy-mediated muscle protein breakdown may contribute to cachexia in breast cancer.

### Mitochondrial dysfunction and impaired glucose handling

Mitochondrial dysfunction in muscle has been reported as a consequence of breast cancer metastasis (Fig. 2). In mice inoculated with human MDA-MB-231 breast cancer cells, expression of proteins in muscle involved in mitochondrial biogenesis was decreased [104]. The same study also observed increased autophagy-related protein expression in mitochondrial fractions of muscle [104], indicating an increase in mitophagy. Further evidence of mitochondrial deficits in breast cancer cachectic muscle was demonstrated in a study that compared isolated skeletal muscle fibers from 13 breast cancer patients to those of healthy females, revealing decreased mitochondrial content in both intermyofibrillar and subsarcolemmal regions of muscles, measured by electron microscopy [48].

CM from breast cancer cells can also impair glucose uptake into skeletal muscle cells *in vitro* (Fig. 2). Treatment of rat myotubes with CM from either MCF-7 or BT474 cells disrupted the translocation of glucose transporter 4 (GLUT4) to the plasma membrane, resulting in reduced glucose uptake into skeletal muscle cells relative to treatment with CM from the non-tumorigenic MCF-10A line [106]. Furthermore, insulin signaling though Rac1 was diminished in this experimental model [106]. These data indicate that impaired glucose uptake into skeletal muscle may be another mechanism causing muscle weakness in breast cancer metastasis.

### Impaired ion transport into muscle cells

As previously discussed, Waning and colleagues demonstrated that muscle proteins of mice bearing MDA-MB-231 tumors were highly oxidized [84]. In particular the RyR1 Ca^2+^ release channel was highly post-translationally modified [84]. Oxidation of RyR1 channels in skeletal muscle results in a pathological sarcoplasmic reticulum (SR) Ca^2+^ leak, which is associated with muscle weakness [84] (Fig. 2). Waning and colleagues followed up these results in muscle from breast cancer patients with bone metastases and found similar alterations in the RyR1 channel [84]. Therefore, impairments in muscle function in cachexia may be a consequence impaired Ca^2+^ handling.

Another mechanism of muscle dysfunction described in the literature is impaired transport of ions within muscle. Wang and colleagues identified changes in genes associated with Zinc ion binding in muscles of mice bearing 4T1 (breast) and C26m2 (colon) tumors through RNAseq analysis [78]. The authors focused on the metal transporter *Zip14*, as it was highly expressed in both tibialis anterior and diaphragm muscles in tumor-bearing mice relative to the respective controls [78]. Further analysis revealed that Zip14 induction was induced by TNF-α and TGF-β, as neutralization of these cytokines with antibodies prevented enhanced Zip14 expression [78] (Fig. 2). Knockout of Zip14 also reversed evidence of cachexia in mice, as measured by an increase in body weight, muscle CSA, and a reversal of the increase in E3 ligase gene expression in 4T1 tumor-bearing mice [78]. This increase in the metal transporter resulted in increased zinc, iron, manganese and copper in the gastrocnemius muscle and zinc and iron in the diaphragm [103]. Elevated levels of iron may cause muscle dysfunction through mechanisms such as ferroptosis of muscle cells [107].

### Immune cell infiltration into muscle

The infiltration of CD45^+^ immune cells into muscle has been observed in multiple models of cachexia, including colon, lung and pancreatic cancer [108], and more recently breast cancer [79]. Neyroud and colleagues demonstrated that the presence of CD45^+^ immune cells increased in the diaphragm of KPC pancreatic tumor-bearing mice as the disease advanced [108]. A similar phenomenon of increased CD45^+^ cells in tibialis anterior muscles of both 4T1.2 and EMT6.5 breast tumor-bearing mice was also observed in our study [79] (Fig. 2). An increase in neutrophils, monocytes, macrophages and myeloid-derived suppressor cells, but a decrease in B cells and CD4 T cells was found in C26 tumor-bearing mice relatively to sham and weight stable mice [109]. In support, increased CD11b^+^ myeloid cells were found in cachectic, pancreatic ductal carcinoma (PDAC) patients relative to weight stable and healthy individuals [110]. Similarly, in transgenic KPP pancreatic tumor-bearing mice and LLC lung tumor-bearing mice, macrophages, neutrophils and eosinophils in LLC but not KPP were elevated in hindlimb muscles, as measured by flow cytometry [111].

In breast cancer models, monocytes are elevated in the circulation [112, 113]. In 4T1 tumor-bearing mice, chemokine (C–C motif) ligand 2 (CCL2) was shown to promote cachexia [102], possibly through chemoattraction of immune cells (Fig. 2). In mice administered 4T1 cells through intravenous injection, circulating IL36G expressing neutrophil-like cells were reported to promote cachexia [112]. Furthermore, we have demonstrated increased Ly6G^+^ cells within muscles of cachectic EMT6.5 breast tumor-bearing mice [79]. These data indicate that myeloid-derived immune cells may be promoting cachexia in breast cancer. Neutrophil levels have also been reported to be elevated in blood, lung and liver of cachectic C26 and KPC tumor-bearing mice [112–114]. This observation is consistent with data collected from patients with metastatic colon, prostate and lung cancers, where an elevated neutrophil-to-lymphocyte ratio has been found in blood samples [114]. Neutrophil-to-lymphocyte ratio relative to hand-grip strength can be used to predict cancer outcomes [115]. Targeting neutrophils may be a viable therapeutic strategy to negate cachexia, as depletion of Ly6G^+^ cells reduced muscle wasting in a mouse model of pancreatic cancer [116]. Targeting other myeloid cells, such as macrophages has also been demonstrated to reduce cachexia [73, 110, 115, 117]; however, the role of macrophages in breast cancer-induced cachexia is not well established.

### Alterations in the neuromuscular junction architecture

Changes in the neuromuscular junction (NMJ) have been described as a driver of cachexia, with perturbations in the structure of the motor unit observed in both patient samples and in C26 colon tumor-bearing mice [118]. Single nuclear RNA sequencing of muscles from mice with pancreatic cancer revealed that genes associated with NMJ remodeling were elevated [119]. In both 4T1.2 and EMT6.5 breast tumor-bearing mice, the area of the motor endplate was reduced compared to sham mice [79] (Fig. 2). These morphological changes were associated with altered expression of structures and genes, consistent with other studies that report deficits in the NMJ in cachexia [120]. However, in contrast to previous reports in other cancers [118], there was no deficit observed in the structure of the motor nerve itself in 4T1.2 and EMT6.5 tumor-bearing mice [79].

## Therapy-induced cachexia in breast cancer

Anti-cancer therapies can also contribute to the onset and progression of cachexia [121] (Fig. 2), with chemotherapy well-documented to cause muscle and fat loss [122]. A direct effect of chemotherapy on muscle cells was demonstrated by treating differentiated C2C12 myotubes with doxorubicin for 48 h, which resulted in an increase in reactive oxygen species (ROS) and smaller myotubes *in vitro* [123]. In mice, doxorubicin was shown to accumulate in the mitochondria, an event that could be reversed by treadmill running [124]. A transcriptional analysis of muscle from C57/BL6 mice treated with doxorubicin revealed an increase in p53/p21 pathways and the induction of DNA damage [125]. 5-FU treatment of Balb/c mice bearing C26 tumors resulted in reduced ERK signaling [126], which regulates muscle mass and slow-programming of skeletal muscle [127]. Slow-programming of skeletal muscle has been shown to be protective against force deficits in mouse models of muscle dystrophy [128], therefore suggesting that disruption of ERK signaling through chemotherapy may shift muscle to a fast fiber phenotype and therefore may be more susceptible to muscle loss. Pin and colleagues also showed that cancer and chemotherapy both shared and displayed unique alterations in muscle energy metabolism [129]. Mice administered chemotherapy also displayed reductions in NMJ health as measured by electromyography (EMG), and through western blotting for markers of the motor endplate such as Musk, Lrp4, Dok7 and Rapsyn [120]. Other causes of muscle atrophy following chemotherapy, such as an increase in inflammatory cytokines with associated elevation of markers of protein ubiquitination and autophagy have been described [130].

Chemotherapy-induced cachexia has been assessed in breast cancer models. The inoculation of MDA-MB-231 cells into the left ventricle of the heart of nude mice, or treatment with carboplatin resulted in wasting and weakness in the mice [131]. Combination treatment of both tumor cells and carboplatin did not have an additive effect, and cachexia severity remained similar to the MDA-MB-231-only group, despite a reduced tumor burden achieved with the therapy [131]. Administration of an aromatase inhibitor to nude mice reduced specific force of the extensor digitorum longus muscle [132]. In Balb/c mice injected with 4T1 cells into the flank, both tumor-bearing and paclitaxel chemotherapy reduced muscle and fat mass relative to non-tumor-bearing mice and once again, there was no additive effect of chemotherapy administration [133]. These studies demonstrate that chemotherapy induces muscle wasting in mice with breast cancer. The mechanisms may be distinct from tumor-induced muscle wasting and further studies are required to tease out the mechanisms behind these responses (Fig. 2). It is also important to note that chemotherapy agents can also reduce appetite [134], which may compound any direct effects on muscle strength and mass.

Radiation therapy may also contribute to cachexia progression in breast cancer (Fig. 2). In head and neck squamous cell carcinoma patients, lumbar skeletal muscle mass decreased during the course of radiotherapy and chemoradiotherapy [135]. In nasopharyngeal carcinoma patients, the mean weight loss during radiotherapy was 6.85% [136]. As reviewed by Pwrózek and colleagues, radiation therapy can lead to damage of structures in the digestive tract and sensory neurons that may alter food intake for cancer patients [137]. Radiation therapy can also alter taste and contribute to a loss of appetite, further exacerbating weight loss [137]. A study of pectoralis muscle quality measured by ultrasound, revealed that breast cancer survivors who had received radiation therapy either to the breast only, or to the breast and axillary lymph nodes had reduced muscle quality compared to healthy volunteers [138]. Furthermore, Wallner and colleagues demonstrated that pectoralis muscles from breast cancer patients who had undergone radiation therapy had increased neutrophil infiltration, more connective tissue and a gene signature indicative of muscle inflammation [139], indicating that radiation therapy may also cause damage to muscles in close proximity to the radiation exposure site (Fig. 2).

It is less clear if other breast cancer therapies, such as immunotherapy, contribute to cachexia progression. PD-1 blockade using nivolumab or pembrolizumab has been reported to cause muscle weakness, myalgia and increased serum creatine kinase levels [140], indicative of muscle damage. Furthermore, immunotherapy is well documented to cause peripheral neuropathy [141] (Fig. 2). As described previously, alterations in peripheral nerves have been reported to promote cancer-associated cachexia [118, 119, 140], highlighting the possibility that immunotherapy may exacerbate this motor neuron pathology. In cancer survivors who have received anti-PD1 therapy, there have been reports of neuromuscular disorders such as myasthenia gravis, immune-mediated myopathies, and Guillain–Barre syndrome [142]. Additionally, as with other treatment modalities, immune checkpoint inhibitors can also negatively affect appetite in cancer patients, which may compound cachexia progression [143]. Therefore, despite the emergence of immunotherapy as an exciting new treatment modality, it is important that patients are monitored closely for myopathies. Therefore, future research should investigate therapies to spare peripheral nerve health during immunotherapy treatment.

## Modelling cachexia in metastatic breast cancer

**Fig. 3:**
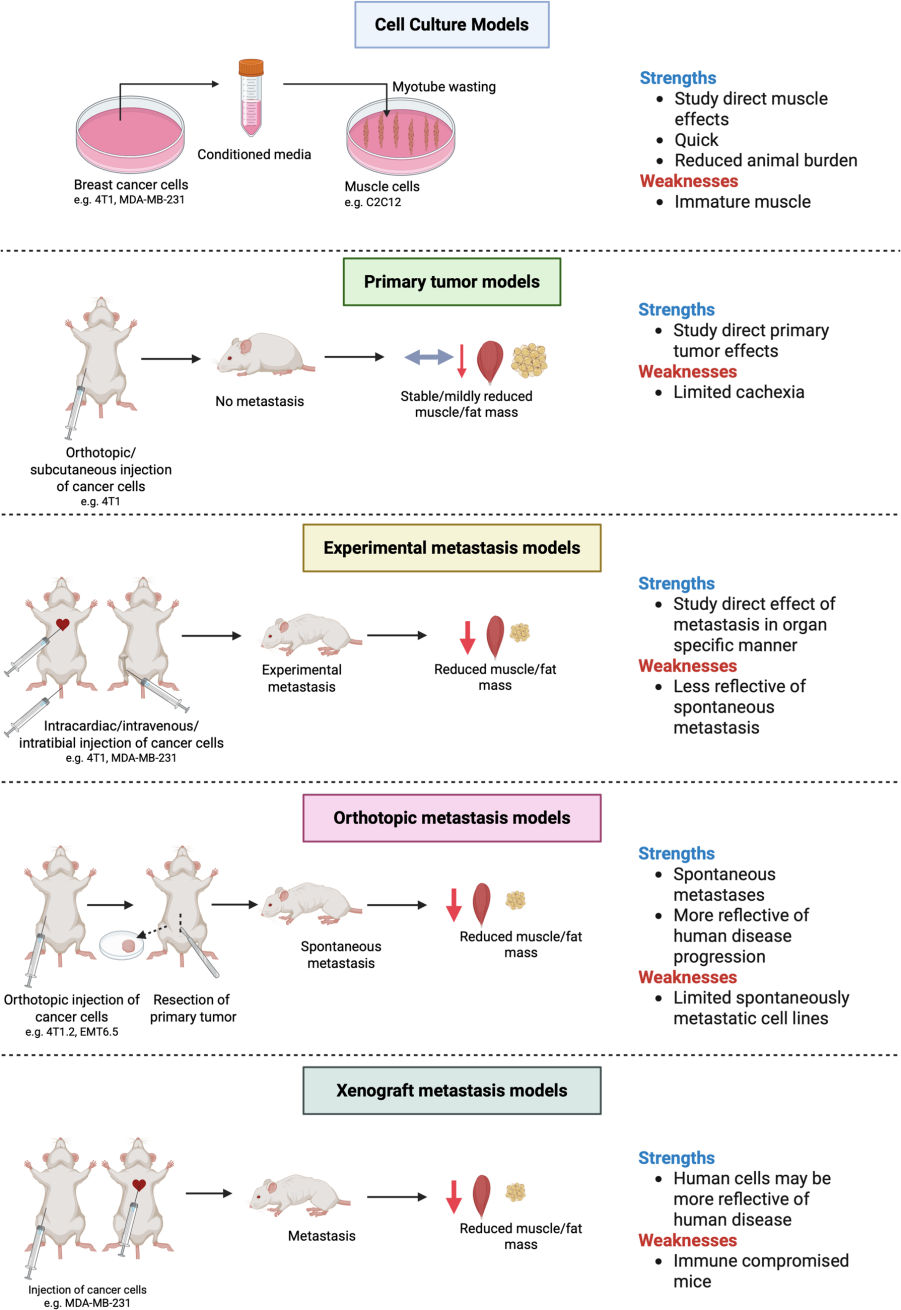
Models of cachexia in breast cancer. Preclinical studies have utilized multiple different models in which to study cachexia in breast cancer, including cell culture, primary tumor, experimental metastasis, orthotopic metastasis and humanized models. The strengths and weaknesses of these models are highlighted on the right side of the figure. Created in BioRender. Saunders, A. (2026) https://BioRender.com/shs4o5d

There have been multiple recent reports of preclinical models to study cachexia in breast cancer (Fig. 3). A review from Tomasin and colleagues discussed the failure of many anti-cachectic agents in clinical trials. They highlighted the models with diverse age, presence of co-morbidities, chemotherapy administration and the fact that different models may be useful for testing potential new agents [144]. Their review promoted orthotopic tumor implantation and spontaneous metastatic models, to improve translatability. In the following section we review the current mouse models used to model cachexia in breast cancer.

In cell culture, CM from 4T1 tumor cells increased protein expression of E3 ligases such as Atrogin-1 and Murf-1, as well as LC3B in C2C12 myotubes as measured by western blotting [102, 145]. 4T1 CM can also induce apoptosis in C2C12 myotubes, as revealed by elevated cleaved caspase-3 [102]. Similar results were observed following treatment of C2C12 muscle cells with CM from MDA-MB-231 cells [102]. While these data indicate that *in vitro* models may be useful screening tools to test mechanisms of cachexia, it is important to note that muscle cells in culture are relatively immature compared to mammalian skeletal muscle. Cells in culture are also devoid of cues from the motor nerve, infiltrating immune cells, a blood supply and crosstalk between other organs. Therefore, it is important that *in vivo* models are also used to accurately recapitulate cachexia biology.

Multiple studies have utilized models of primary breast tumors to study cachexia (Fig. 3, Table 3). As summarized in Table 3, mice engrafted orthotopically with MCF-7 cells develop only 5% body mass loss, with no reported lean or fat mass loss [55]. Engraftment of 4T1 cells into mice induces significant fat mass loss (Table 3), and between 22–38% muscle mass loss [146, 147] (Table 3). However, another study reported no change in fat or lean mass in the 4T1 model [148]. These variable results in primary tumor models may reflect the relatively low prevalence of cachexia in breast cancer patients, where primary tumor cues alone may not frequently induce cachexia.

**Table 3:** Summary of cachexia studies in breast cancer models

Model	Administration route	Subtype	Surgery	Body mass loss	Fat mass loss	Muscle mass loss	Myotube wasting (*in vitro*)	Example Reference
Breast cancer CM	Conditioned media	TNBC	-	-	-	-	−64%	PMID: 38973385 [102] PMID: 41042902 [106] PMID: 40810552 [145]
4T1 EV	EV	TNBC	-	N.R	~ 40–70%	N.R	-	PMID: 37620316 [67]
MDA-MB-231 EV	EV	TNBC	-	N.R	~ 30–80%	N.R	-	PMID: 37620316 [67]
MCF-7 EV	EV	Luminal A	-	N.R	N.D	N.R	-	PMID: 37620316 [67]
PDX	Orthotopic	-	-	N.R	N.R	N.D	-	PMID: 30559167 [53] PMID: 38651826 [54]
MCF-7	Orthotopic	Luminal A	-	~ 5%	N.R	N.R	-	PMID: 14633625 [55]
PyMT	Transgenic mouse	Luminal B	-	~ + 30%	~ 50%	~ + 5–10%	-	PMID: 28978719 [149] PMID: 33842876 [150]
4T1	Flank	TNBC	-	N.D.- ~ + 9%	N.D.- ~ 82%	N.D.- ~ 22–38%	-	PMID: 27863332 [146] PMID: 35132306 [147] PMID: 27212031 [148]
MDA-MB-231	Intracardiac	TNBC	-	~ 20%	~ 25%	N.D.- ~ 20%	-	PMID: 26457758 [84]
4T1	Intravenous	TNBC	-	~ 15%	N.R	~ 15%	-	PMID: 39266531 [112]
4T1	Intratibial	TNBC	-	~ 25%	~ 50%	~ 15%−35%	-	PMID: 29312148 [87]
4T1	Orthotopic	TNBC	-	~ 10%	~ 38%	N.D	-	PMID: 38973385 [102]
4T1	Orthotopic	TNBC	Resected	~ 25%	N.R	N.R	-	PMID: 29875463 [78]
4T1.2	Orthotopic	TNBC	Resected	N.D	~ 30%	~ 9–10%	-	PMID: 41042733 [79]
Bard1	Transgenic mouse	TNBC	-	~ 15%	N.R	N.R	-	PMID: 32730698 [103]
MDA-MB-231-HM	Orthotopic	TNBC	Resected	~ 6.3%	~ 63%	~ 13–16%	-	PMID: 41042733 [79]
EMT6.5	Orthotopic	TNBC	Resected	~ 18.3%	~ 75%	~ 15–17%	-	PMID: 41042733 [79]

- = not reported in study, *N.R.* Not reported. *N.D.* Not different. *PDX* patient-derived xenograft *TNBC* triple-negative breast cancer. *CM* Conditioned media. *EV* Extracellular vesicle

Experimental or ectopic models of metastasis have been utilized to model cachexia. For instance, intracardiac injection of MDA-MB-231 cells results in lean mass and fat mass loss [84] (Table 3), while intratibial injection of 4T1 cells results in muscle atrophy in the opposite leg [87]. There are distinct advantages of these experimental models that limit metastatic tumor growth to a specific organ (*e.g.* bone), which may uncover organ-specific cachectic cues (Fig. 3). A limitation of these experimental metastatic models is that cues that allow cells to mobilize from the primary site, survive in the circulation and develop a metastatic niche for subsequent colonization are not considered. We therefore utilized three spontaneously metastatic models. Orthotopic injection of 4T1.2 cells followed by tumor resection led to a 30% loss of fat mass, and ~ 10% muscle mass loss [79] (Table 3) [79]. A similar response was observed when 4T1 primary tumors were resected to allow for metastasis development [78]. Administration of EMT6.5 cells *via* the same regimen resulted in a 75% loss of fat mass and a 15–17% loss of muscle mass [79] (Table 3). In transgenic PyMT mice muscle atrophy of 5–10% has been observed [149, 150] (Table 3). Thus, orthotopic models of breast cancer can recapitulate the diversity of cachexia seen in breast cancer patients. Xenograft models have also been utilized to study cachexia. Studies such as those conducted by Waning and colleagues have shown that intracardiac injection of MDA-MB-231 cells results in approximately 15–20% loss in body and muscle mass [84, 104]. Similarly with the replication of cachexia in immune-competent mice with orthotopic injection *vs*. experimental metastatic models, we have also shown that orthotopic injection of MDA-MB-231-HM cells results in approximately 15% loss of muscle mass [79].

**Fig. 4:**
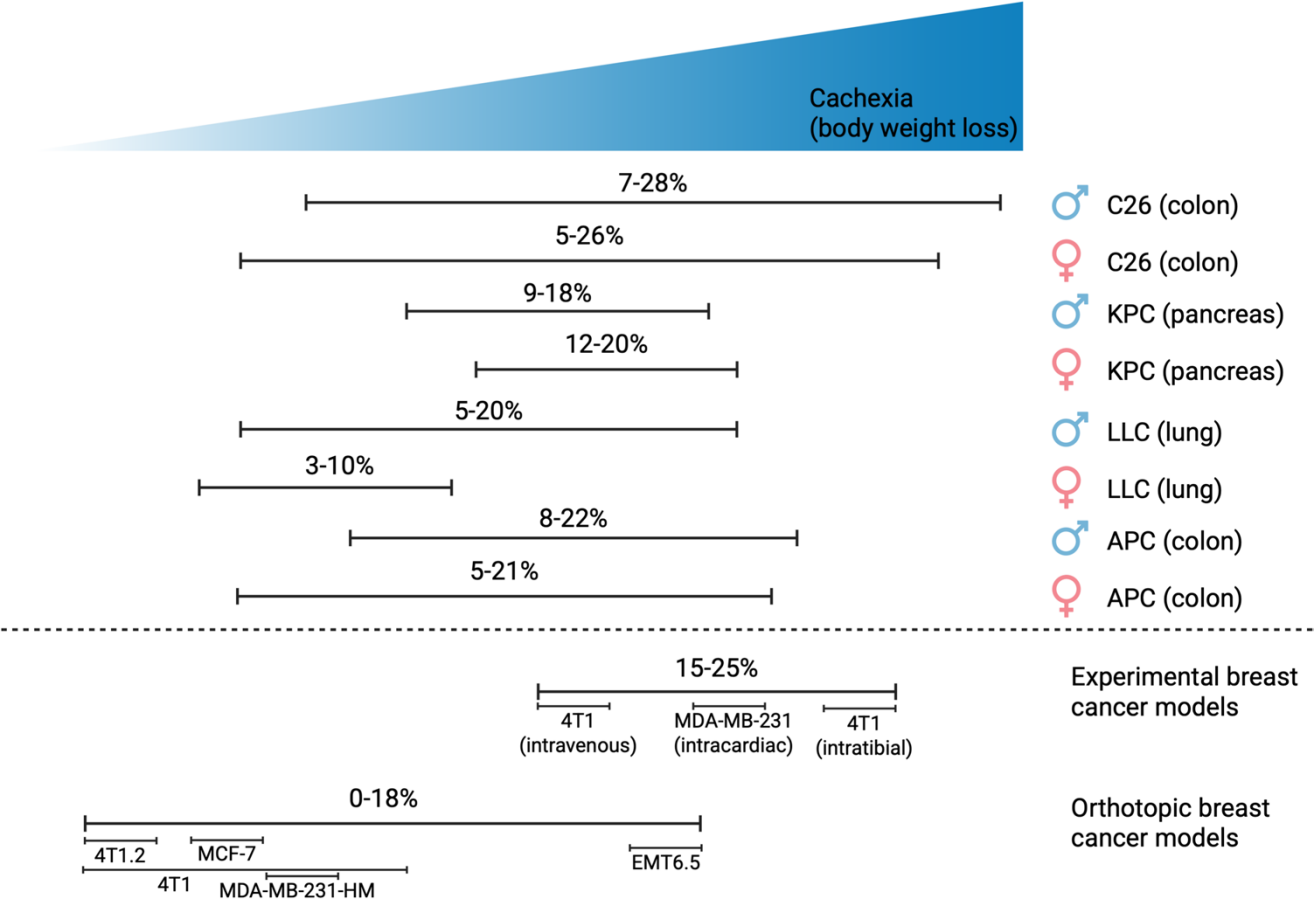
Comparison of cachexia incidence across preclinical breast cancer models and common cachexia models. Values expressed as percentage body weight loss. Degree of cachexia in non-breast cancer models gathered from Morena et al., [43]*.* Created in BioRender. Saunders, A. (2026) https://BioRender.com/6lma97d

In order to put the cachexia in these models in context, we compared the severity of cachexia to four models of cachexia. Morena and colleagues collated cachexia severity in C26, KPC, LLC and APC models in both male and females mice [151]. The values provided by the Morena et al., review were used as a reference to compare the degrees of body mass loss (Fig. 4).

## Conclusions and future directions

Although cancer rarely colonizes skeletal muscle [152], the health of these muscles can be significantly reduced in the setting of cancer. While cachexia in breast cancer does not occur as frequently as in other cancer types such as pancreatic, digestive and lung cancers, approximately a quarter of breast cancer patients will develop this debilitating condition, significantly impacting quality of life and response to cancer therapies. The prevalence is likely to be higher in patients with metastatic breast cancer, with estimates up to 50% of patients [5, 40]. Furthermore, when taking into account the frequency of breast cancer, the incidence of cachexia becomes significant. It is therefore important that breast cancer is not overlooked in discussions of cachexia and we recommend the use of preclinical models that accurately recapitulate the biology of metastasis in breast cancer patients. There are a number of preclinical studies of cachexia in breast cancer, using primary cancer models, [146–148], experimental metastasis [87] and orthotopic models [79] (Fig. 3).

We have summarized the results from preclinical models in current use to investigate mechanisms of muscle weakness and wasting in breast cancer. Primary breast tumor models [55, 146–150], and experimental metastatic models (*e.g.* injection into the circulation or directly in metastatic sites) [84, 87] have the advantage of localization of tumor cells to one or few organs, but they do not reflect the biology of metastasis in patients. Recently, spontaneous metastasis models arising from an orthotopic primary tumor that may be more reflective of metastatic biology [153] have been utilized [78, 79]. We recommend that future studies take advantage of orthotopic models where the primary tumor is localized in the mammary gland and spontaneous metastases develop in different organs.

Future studies should further investigate the mechanisms leading to muscle weakness and wasting caused by existing standard of care therapies, such as chemotherapy. Clinical and preclinical studies should also consider analysis of muscle function and mass following emerging therapies for breast cancer such as CDK4/6 inhibitors, PARP inhibitors, immunotherapy and CAR-T cell therapies. Emergence of more targeted therapies such as antibody–drug conjugates will hopefully lead to reduced toxicity and cause fewer muscle deficits [154]. However, more research is required on this topic, as nonspecific uptake of the antibody–drug conjugates in peripheral nerves is documented, which can lead to inhibition of axonal firing and degeneration of peripheral nerves [155, 156], which can manifest as muscle weakness [155].

Factors secreted from different metastatic lesions that contribute to cachexia in breast cancer should be another area of active investigation. For example, do patients with liver or lung metastases have similar cachectic pathology? Secretion of cytokines such as TGF-β from bone metastatic lesions has been well characterized in preclinical models, however the signals from other metastatic lesions are not well resolved. A comparison of the cachectic factors secreted from more mild models of breast cancer cachexia (such as 4T1) with those from more severely cachectic models (such as EMT6.5) will help to uncover novel regulators of cachexia in metastatic cancer. Understanding these unique atrophy-inducing cytokines may lead to the discovery of novel therapeutic targets that could be tested preclinically/clinically and could lead to more personalized therapies depending on both the type of cancer, and distribution of metastatic lesions present in a patient.

In summary, cachexia affects a significant proportion of breast cancer patients; however, relatively few preclinical studies of cachexia have been published. Mouse models are an essential tool for researchers to understand cancer biology. Complications from cancer such as cachexia occur from the culmination of crosstalk between tumor cells, visceral organs, fat and muscle [157]. As reviewed herein, orthotopic models of breast cancer may provide a novel tool to understand the mechanisms of cachexia in the context of metastasis. Effective therapies and interventions against cancer cachexia have the potential to improve response to cancer therapy, and to improve the quality of life of metastatic cancer patients, highlighting the importance of understanding the drivers of cachexia in the context of metastatic breast cancer.

## Supplementary Information

Below is the link to the electronic supplementary material.

Supplementary file1 (XLSX 13 KB)

## Data Availability

No datasets were generated or analysed during the current study.
